# BaSDAS: a web-based pooled CRISPR-Cas9 knockout screening data analysis system

**DOI:** 10.5808/GI.2020.18.4.e46

**Published:** 2020-12-11

**Authors:** Young-Kyu Park, Byoung-Ha Yoon, Seung-Jin Park, Byung Kwon Kim, Seon-Young Kim

**Affiliations:** 1 OmicsPia Co., Ltd., Daejeon 34867, Korea; 2 Personalized Genomic Medicine Research Center, Korea Research Institute of Bioscience and Biotechnology (KRIBB), Daejeon 34141, Korea; 3 Department of Functional Genomics, University of Science and Technology (UST), Daejeon 34113, Korea

**Keywords:** CRISPR-Cas Systems, data visualization, gene editing, gene expression, pathway analysis

## Abstract

We developed the BaSDAS (Barcode-Seq Data Analysis System), a GUI-based pooled knockout screening data analysis system, to facilitate the analysis of pooled knockout screen data easily and effectively by researchers with limited bioinformatics skills. The BaSDAS supports the analysis of various pooled screening libraries, including yeast, human, and mouse libraries, and provides many useful statistical and visualization functions with a user-friendly web interface for convenience. We expect that BaSDAS will be a useful tool for the analysis of genome-wide screening data and will support the development of novel drugs based on functional genomics information.

**Availability:** BasDAS is freely available at http://barcode.appex.kr/barcode.

## Introduction

As a means for the functional study of genes at the genome-wide level, a genome-wide screening method based on the loss of function of genes is useful. Various methods have been developed for this purpose, including random mutagenesis-based gene knockout, RNA interference-based knockdown, homologous recombination-based deletion with barcode technology, and CRISPR/Cas9-based knockout [1-6]. With a careful design of genome-wide barcodes and decoding of barcode information by next-generation sequencing, those tools and libraries have been widely used to characterize gene function and to discover novel drug targets in various organisms.

As genome-wide barcode screening tools have been widely used, many algorithms and tools have been developed to help researchers to analyze their genome-wide barcode screening data [6-8]. Among the most widely used tools are MAGeCK and MAGeCKflute, which provide a comprehensive suite of tools from quality control to data analysis and visualization using the R statistical language system [6-8]. While useful, those tools are not easy to use for many researchers with limited bioinformatics skills, as they were developed to operate on a command line basis in the Linux operating system. Therefore, it is necessary to develop a tool that can be easily accessed by general researchers who perform functional genomics research using genome-wide pooled screening data. Here, we present BaSDAS (Barcode-Seq Data Analysis System) as a user-friendly web service that is useful for the analysis of genome-wide pooled barcode screening data using next-generation sequencing technology.

## Pipeline Overview

BaSDAS is an automated pipeline that analyzes CRISPR-Cas9 knockout screening data in four steps: (1) data input, (2) primary analysis using the MAGeCK algorithm [7], (3) downstream analysis and visualization, and (4) generation of output.

**Fig. 1.**
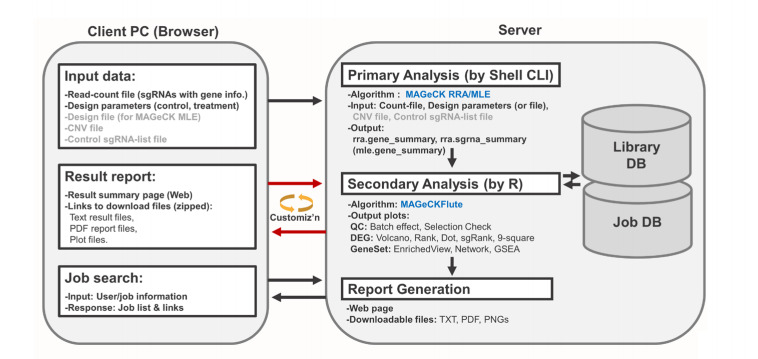
Scheme of the CRISPR-Cas9 knockout screening data analysis system.

We used PHP for the creation of the web interface, R for the data analysis at the server side, Python for data handling, and a MySQL database for the effective management of data and analysis jobs of multiple users (Fig. 1).

The BaSDAS system receives read-count file and analysis parameters as input data and then processes the data by using MAGeCK’s robust ranking aggregation or maximum likelihood estimation algorithm [7] depending on the experimental design. The required parameters include the type of experiment design and source organism information (Supplementary Fig. 1). The counts of the sgRNA or barcode sequence are analyzed in the primary analysis steps, and the results are used for various secondary analyses including plots of negative and positive selection of genes, enrichment analysis, and visualization [8].

## Analysis of Data

**Fig. 2.**
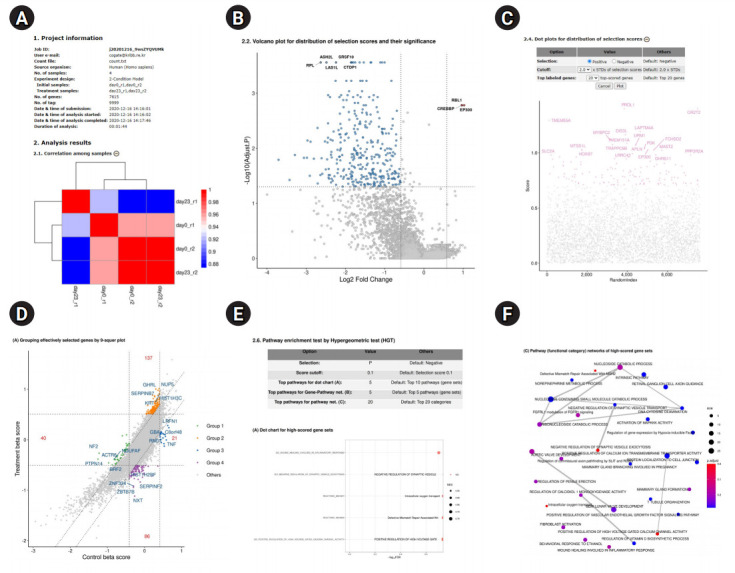
Overview of BaSDAS analysis results. (A) The first part of the analysis report page. “Project information” provides the following information: job ID, e-mail, input tag-count file, number of samples, experimental model, sample assignment to the conditions, number of knockout tags, running time of analysis process, etc. (refer to the upper part of Fig. 2A). (B) Volcano plot for the distribution of selection scores and their significance. (‒) buttons (when the plot appears) and (+) buttons (when the plot disappears) are provided on the right end of the plot titles. These buttons act as toggles to show or hide the plots. This function is helpful to understand the overall structure of the entire report by reducing the number of pages of the report. (C) Parameter setup table for redrawing dot plots of gene selection scores. Changing or modifying the parameters for reanalysis and replotting: the parameter values can be reset or modified if needed. (D) Grouping of selected genes and pathway analysis. (E) Pathway enrichment test by the hypergeometric test. (F) Pathway (functional category) networks of highly scored gene sets.

The main functions of the BaSDAS system are (1) analyzing positively or negatively selected genes with the user tag-read count file from genome-wide screening experiments as a primary analysis (Fig. 2B and C) and (2) conducting a secondary in-depth analysis to estimate the molecular functions or pathways to which the selected gene groups belong (Fig. 2D-2F). The analysis of the user gene knockout screening data is conducted through the following procedure (Supplementary Fig. 1): (1) clicking on the menu item “Analysis,” (2) selecting an experiment model, (3) selecting a source organism, (4) entering the user’s e-mail address, (5) selecting and uploading the user data file (read-count file), (6) selecting the samples for each condition group, (7) submitting the analysis job, (8) monitoring the analysis process, and (9) completing the analysis.

## Generation of the Analysis Results

After the completion of the analysis, users can access to the analysis report in one of the following three ways: (1) by clicking the hyperlink to the analysis report provided on the job monitoring page, (2) by clicking the hyperlink to the analysis report provided in the results table of the job search function, or (3) by clicking the hyperlink to the analysis report in the e-mail reporting that the analysis is complete. Any of those links lead to the analysis report page shown in Fig. 2. The content of the analysis report depends on the selected experimental models. The results of the in-depth analysis and visualization can be revised or modified by repeated reanalyses with various parameters. The total analysis results, from the initial analysis or reanalysis, can be downloaded as a single compressed file through the user’s web browser. The compressed report file includes (1) primary gene selection results, (2) the results of an in-depth analysis and their visualization, and (3) a report in PDF or HTML format.

## Revision of the In-depth Analysis Results

Various plots are given in the analysis report to help users to understand the results through an intuitive visualization. If it is necessary to revise the plotting range or the content of a plot, the user can proceed to re-analyze and plot the data repeatedly by modifying the parameters of the plots or analysis (Fig. 2C). The re-analyzed results and graphs are included in the final analysis report as PDF or HTML files and finally provided as a compressed file. For re-analyzable graphs, the graph editing buttons are provided at the right end of the graph title in the analysis report page. By clicking the graph editing buttons, users can access the parameter-setting table for reanalysis and plot the graphs again. In the table, parameter values used for the current results and plots are listed and the input fields for the parameters—cutoff values, maximum number of genes or pathways, positive or negative selection, and so on—are provided to reset or change the parameters.

## Future Work

Our ultimate goal is to construct a comprehensive analysis environment for the comparative analysis and interpretation of genome-wide pooled screening experiments by building a database of various public pooled CRISPR-Cas9 screening data. Toward this goal, we will collect public genome-wide screening datasets from diverse organisms, construct a database, and also update the comparative analysis modules.

## Conclusion

We have developed a tool that allows researchers with limited bioinformatics skills to easily and effectively analyze their pooled genome-wide screening data. Our system provides many useful functions such as quality control, median normalization, sgRNA mean-variance modeling, sgRNA ranking, and identification of essential genes and enriched pathways from the knockout tag read sequences obtained by genome-wide barcode screening experiments. By developing a GUI-based interface, user convenience is maximized, and various secondary statistical analyses and visualization functions are provided for an intuitive interpretation of the given results. In the future, we plan to build a comprehensive analysis environment for comparative analysis and downstream research by collecting public pooled CRISPR-Cas9 knockout screening data and analysis results and converting them into a database in this BaSDAS system. We hope that BaSDAS will provide researchers a useful tool to effectively analyze and interpret their data to support the development of novel drugs based on functional genomics information.

## Supplementary Materials

Supplementary Figure 1. Analysis of the user gene knockout screening data. (A) Snapshot for user data analysis page after clicking ‘Analysis’ menu. (B) Showing sample selection table after uploading user count file with the progress-bar.
