# Supplementary material for: BaSDAS: a web-based pooled CRISPR-Cas9 knockout screening data analysis system
Source: Genomics Inform. 2020 Dec 11;18(4):e46. doi: 10.5808/GI.2020.18.4.e46 (PMC7808871; doi:10.5808/GI.2020.18.4.e46)
Supplement: Supplementary Figure 1. — Analysis of the user gene knockout screening data. (A) Snapshot for user data analysis page after clicking ‘Analysis’ menu. (B) Showing sample selection table after uploading user count file with the progress-bar. [file gi-2020-18-4-e46-suppl.pdf]

Supplementary Fig. 1

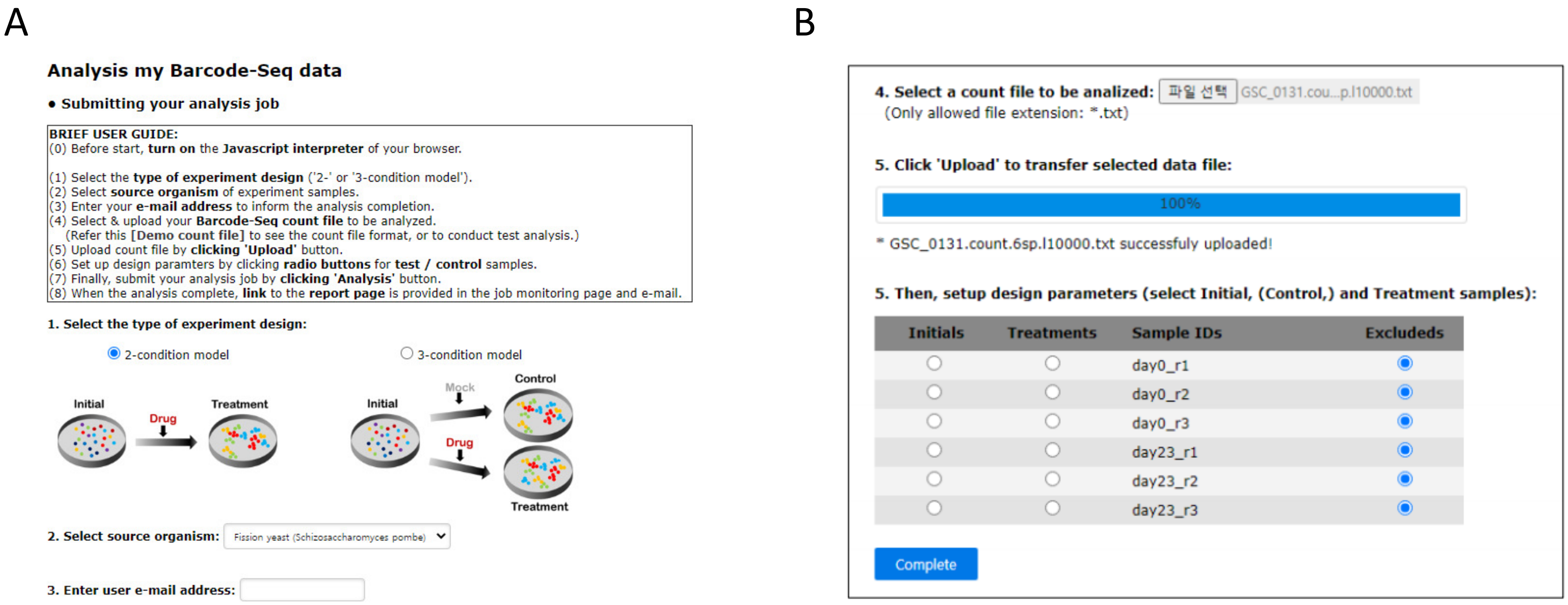

Supplementary Figure 1. Analysis of the user gene knockout screening data. (A) Snapshot for user data analysis page after clicking 'Analysis' menu. (B) Showing sample selection table after uploading user count file with the progress-bar.
